# First evidence for an association between joint hypermobility and excitability in a non-human species, the domestic dog

**DOI:** 10.1038/s41598-019-45096-0

**Published:** 2019-06-13

**Authors:** Jonathan Bowen, Jaume Fatjó, James A. Serpell, Andrea Bulbena-Cabré, Eldin Leighton, Antoni Bulbena

**Affiliations:** 1 The Royal Veterinary College, University of London, London, UK; 2 Department of Psychiatry and Forensic Medicine (Chair Affinity Foundation Animals and Health), Autonomous University of Barcelona, Barcelona, Spain; 3 IMIM-Mar Health Park, Neuropsychiatry and Drug Addiction Institute (INAD), Barcelona, Spain; 4 School of Veterinary Medicine, University of Pennsylvania, Philadelphia, Pennsylvania USA; 5 Department of Psychiatry, Icahn School of Medicine at Mount Sinai, New York, USA; 6 Mental Illness, Research, Education, and clinical Center (MIRECC), James J. Peters VA Medical Center, Bronx, New York, USA; 7 The Seeing Eye, Morristown, New Jersey USA; 8 Centro de Investigación Biomédica en Red de Salud Mental (CIBERSAM), Barcelona, Spain

**Keywords:** Emotion, Sensory processing

## Abstract

There is a well-established relationship between joint hypermobility and anxiety in humans, that has not previously been investigated in other species. A population of 5575 assistance dogs were scored for both hip hypermobility and 13 behaviour characteristics using previously validated methods. Our results suggest a positive association between hip joint hypermobility and emotional arousal in domestic dogs, which parallel results found in people.

## Introduction

Since our first observation of the strong association between anxiety and joint hypermobility^1^, in people, a number of additional studies in clinical and non-clinical groups have supported the association between joint hypermobility syndrome (JHS) and anxiety, and with underlying features such as affective reactivity^2–6^. A systematic review by Sinibaldi, *et al*.^7^ and a review and meta-analysis by Smith, *et al*.^8^ have summarised the clinical associations.

Human patients with JHS exhibit significantly greater manifestations of fear, agoraphobia and panic^4^. Although the strongest associations were initially found to be between JHS and anxiety disorders, recent studies have found similar links with a much broader range of psychiatric conditions^4,5,9^.

Suggested mechanisms in people include indirect effects on emotional and mental state through dysregulation of autonomic responses^5^, involving brain regions such as the insular cortex, amygdala and anterior cingulate cortex, which are linked to increased autonomic arousal that intensifies emotional states^6^. Also, it has been proposed that anxiety could be linked to pain and a greater perception of joint instability in human patients with JHS^8^.

Here we present the first evidence for an association between hip joint hypermobility and behavioural arousal in a non-human species. This suggests that the link between hypermobility and behaviour regulation is phylogenetically old and could potentially be a universal trend among mammals. It has been suggested in the literature that a human patient’s conscious awareness of the negative consequences of joint instability for their health might be the primary source of their stress and anxiety. The findings of the current study challenge that interpretation.

We selected the domestic dog (*Canis familiaris*) as a candidate species as it presents physical health problems related to JHS and experiences naturally occurring behavioural disorders that show face validity, and potential homology, with previously defined psychiatric disorders in humans^10^. Human behavioural characteristics that could represent transdiagnostic traits across a range of psychiatric condition, such as impulsivity, have also been investigated in dogs^11^.

Hip joint hypermobility is a risk factor for canine hip dysplasia (CHD), one of the most prevalent developmental orthopaedic diseases in dogs worldwide. In one of the most widely used systems for hip evaluation (PennHip), radiographs of the hip joint are taken under extension and the extent to which the head of the femur can be displaced from the acetabulum is measured^12^. This form of hip joint evaluation does not involve assessment of the conformation or structure of the hip; only extension of the joint under anesthesia. The resulting Distraction Index (DI) is therefore primarily a measure of the extensibility of the ligament of the head of the femur (ligamentum teres), and therefore of hip joint hypermobility.

The Canine Behavioural Assessment Research Questionnaire (C-BARQ) is a validated questionnaire for the assessment of 13 behavioural characteristics of dogs^13,14^, which has been used to investigate the heritability of behavioural traits in dogs^15^ and to explore the behavioural characteristics of genetically clustered dog breeds^16^. The C-BARQ sub-scales include stranger-directed aggression, owner-directed aggression, dog-directed aggression/fear, familiar dog directed aggression, trainability, chasing, stranger-directed fear, non-social fear, separation-related problems, touch sensitivity, excitability, attachment/attention seeking, and energy.

The Seeing Eye is a philanthropic organisation with a large database of guide dogs that are routinely assessed for both hypermobility of the hip joint and behaviour traits through the use of PennHip and C-BARQ. This provided us an excellent opportunity to study the association between both conditions in a population of 5575 dogs in a non-invasive manner.

We found 4 clusters of dogs based on their DI (see Table 1 for a summary of the PennHip DI scores for the four clusters).

**Table 1:** Maximum, minimum, mean and standard deviation of DI for the four clusters identified in the analysis.

Complete dataset (n = 5575)	Complete dataset (n = 5575)	Complete dataset (n = 5575)	Complete dataset (n = 5575)	Complete dataset (n = 5575)
n	2153	2139	874	409
Mean PennHip DI score	0.29	0.38	0.47	0.62
Max PennHip DI score	0.33	0.42	0.53	0.92
Min PennHip DI score	0.14	0.34	0.43	0.54
Standard deviation	0.03	0.02	0.03	0.07

Binary logistic regression (BLR) allowed us to identify behaviour and demographic factors associated with membership of the two clusters with the highest and the lowest hypermobility scores. Females were 3.66 times more likely to be in the high hypermobility group than males, which is consistent with findings in humans^8^. There were also significant breed associations, with Golden Retrievers being 8.58 times more likely to be in the high hypermobility group, and both Labrador Retrievers and German Shepherds being around 5 times more likely to be in the lower hypermobility group. With regard to behavioural traits, higher scores for excitability and aggression towards familiar dogs were associated with belonging to the high hypermobility cluster (see Table 2 for a summary of the BLR results).

**Table 2:** Summary of results of binary logistic regression model.

	Wald	Significance	Odds ratio	95% confidence interval for odds ratio	
				Lower	Upper
Sex (female)	71.70	<0.0001	3.66	2.71	4.94
Breed: Labrador Retriever	5.05	0.025	0.21	0.05	0.82
Breed: Golden Retriever	9.67	0.002	8.58	2.21	33.27
Breed: German Shepherd	5.22	0.022	0.20	0.05	0.80
Breed: 1st generation Labrador Retriever/German Shepherd cross breed	0.06	0.804	1.19	0.30	4.80
Breed: 2nd generation Labrador Retriever/German Shepherd cross breed	2.31	0.128	0.32	0.08	1.38
Breed: 3rd generation Labrador Retriever/German Shepherd cross breed	3.40	0.065	0.22	0.04	1.10
Aggression toward owner	1.86	0.173	1.77	0.78	4.03
Aggression toward familiar dog	7.44	0.006	1.70	1.16	2.49
Trainability	0.03	0.870	0.97	0.69	1.36
Chase behaviour	1.30	0.254	0.89	0.73	1.09
Non-social fear	0.00	0.971	1.01	0.70	1.45
Touch/Pain sensitivity	1.20	0.273	0.84	0.62	1.14
Excitability	5.38	0.020	1.26	1.04	1.54

Excitability is a fundamental characteristic of emotional responses. Emotional reactions to stimuli consist of two dimensions; valence and arousal. Valence refers to the negative or positive character of the response, and arousal to its intensity. In the method of behavioural assessment used in our study, excitability is an indication of the ease with which an individual enters a state of high emotional arousal in response to an external stimulus or event, regardless of emotional valence. The C-BARQ describes a highly excitable dog as one that shows marked behavioural arousal, including barking or yelping at the slightest disturbance, rushing toward and around any source of excitement, and having difficulty calming down. In humans, increased arousal has been linked to anxiety symptoms even in the face of neutral stimuli^17^. Both in humans and dogs, high levels of arousal seem to impair top-down cognitive inhibition, which in turn has been linked to anxiety^18,19^. All C-BARQ sub-scales are scored 0–4, and within the BLR model, for every 1-point increase in excitability sub-scale score a dog was 1.26 times more likely to be in the high DI group. This finding is in agreement with previous studies in humans that showed that subjects with JHS have an increased sensitivity and reactivity to some sensory modalities such as interoception, pain and visual emotional stimuli. A study comparing hypermobile and healthy patients^3^ found structural, but more important, functional differences in key brain regions involved in emotional processing and arousal, which seem to mediate the link between hypermobility and anxiety^6^. More recently, our group found that patients with JHS and panic disorder exhibited higher olfactory acuity, increased odour reactivity and also greater odour awareness compared to healthy controls^20^. Our results in dogs seem to be in accordance with what has been found in human beings and present excitability as a potential trans diagnostic trait that could be involved in the relationship between joint hypermobility and anxiety.

The only other association between hypermobility and a behaviour dimension was for aggression directed to familiar dogs. This is perhaps unsurprising, as dogs in the Seeing Eye program ought to have a low general prevalence of problem behaviour; they are reared and trained in a manner that is intended to prepare them as thoroughly as possible for their future working role. This is confirmed by the generally lower scores for the C-BARQ sub-scales relating to problem behaviour in the study population compared with scores from a general population of 33,708 dogs from a range of breeds that were obtained from the C-BARQ database compiled by the School of Veterinary Medicine, University of Pennsylvania (see Table 3 for a summary of C-BARQ scores for the study population and a general population). For example, the mean score for stranger-directed fear in the general population is 0.67, compared with 0.05 for the study population. In the BLR model, for every 1-point increase in aggression directed to familiar dogs sub-scale a dog was 1.7 times more likely to be in the high DI group. Aggression towards familiar dogs describes a situation of species-specific social conflict between two or more dogs living in the same household that is unlikely to be strongly influenced by an assistance dog rearing programme. Interestingly, canine intra-group conflict usually includes a dimension of ambivalence and anxiety^21^, which would help to explain the association we found between this form of aggression and joint hypermobility. However, a weakness of the dataset was that the instructions for C-BARQ tell respondents not to complete the block of questions on familiar-dog aggression if there was no other dog in the household. Without other confirmatory information, we could not determine whether respondents really had only one dog in the house, or had simply skipped that set of questions for some other reason. Also, aggression might be directed at familiar dogs that are not resident in the household, and the questionnaire, as it was completed, would miss this.

**Table 3:** Mean C-BARQ scores, with standard deviation, for the study and a general population.

	Trainability	Stranger-directed aggression	Owner-directed aggression	Unfamiliar dog-directed fear and aggression	Familiar-dog aggression	Stranger-directed fear	Non-social fear	Touch sensitivity	Separation-related problems	Excitability	Attention seeking	Chasing	Energy
Mean (general population)	**2.60**	**0.65**	**0.17**	**0.91**	**0.57**	**0.67**	**0.84**	**0.76**	**0.60**	**2.10**	**2.01**	**2.13**	**2.02**
S.D.	0.64	0.73	0.41	0.80	0.78	0.95	0.77	0.79	0.66	0.83	0.81	1.11	1.07
Mean (study population)	**2.88**	**0.11**	**0.06**	**0.26**	**0.14**	**0.05**	**0.33**	**0.44**	**0.37**	**1.76**	**1.78**	**1.18**	**2.37**
S.D.	0.45	0.26	0.16	0.41	0.34	0.21	0.39	0.56	0.40	0.77	0.70	0.81	0.89
Mean (low laxity cluster)	**2.88**	**0.11**	**0.06**	**0.26**	**0.13**	**0.05**	**0.31**	**0.45**	**0.37**	**1.73**	**1.77**	**1.17**	**2.36**
S.D.	0.45	0.26	0.16	0.42	0.34	0.21	0.36	0.56	0.40	0.77	0.70	0.81	0.89
Mean (high laxity cluster)	**2.84**	**0.12**	**0.08**	**0.23**	**0.17**	**0.06**	**0.43**	**0.35**	**0.35**	**1.88**	**1.81**	**1.27**	**2.42**
S.D.	0.46	0.26	0.18	0.37	0.37	0.24	0.51	0.50	0.38	0.76	0.69	0.80	0.91

When interpreting the apparent association between hypermobility and behavioural characteristics found in our results, pain should be considered a potentially influential factor. A PennHip DI score of above 0.3 has been identified as an indicator of increased individual risk of hip dysplasia (HD). Mean DI for clusters 1 and 4 were 0.29 and 0.62 respectively. Cluster 4 dogs would therefore be at greater risk of the development of HD. However, dogs had undergone the assessment of hip distraction at approximately 18 months of age, but usually pain commences at a later age when the joint becomes degenerate or inflamed. Moreover, a dog may have good DI and yet be in pain or vice versa. According to the evidence-based joint-guidelines of the American Animal Hospital Association (AAHA) and American Association of Feline Practitioners (AAFP)^22^, defensiveness and touch/pain sensitivity would be expected to be increased, and activity to be decreased, in dogs that are experiencing chronic or acute pain. However, our BLR model did not reveal differences between high and low hypermobility groups with respect to behaviour traits which would be expected to be influenced by pain, such as touch sensitivity, energy level (as an indicator of activity), or aggression in other situations. Although familiar dog directed aggression might be associated with pain, particularly during play and similar social interactions, the same would not seem to apply to excitability (which would not be expected to be increased in the absence of the other pain indicators such as defensiveness, reduced activity and touch/pain sensitivity). So, we suggest that differences in behaviour between the high and low hypermobility groups cannot be accounted for entirely by pain.

In summary, our results in Seeing-Eye dogs suggest that the link between hypermobility and emotional arousal is not limited to human beings. Also, we hypothesise that overall excitability could be a key underlying trait involved in the development of anxiety-related disorders both in humans and animals. However, since this study involved a selectively bred population of assistance dogs, further work is required in order to determine whether these findings can be generalised to other types of dogs.

## Methods

Data on an initial population of 5602 dogs, which had been collected over the period from 2001 to 2014, was supplied by The Seeing Eye organisation. All dogs had undergone a PennHip assessment of hip distraction at approximately 18 months of age (mean age = 516 days, standard deviation = 69), and a behavioural assessment using the C-BARQ at 12 months of age. The dogs lived with their foster families from 2 until at least 14 months of age; C-BARQ was completed for each dog by the member of the foster family who had primary responsibility for the dog (and was therefore very familiar with the dog).

The following breeds were excluded as they represented only very small proportions of the population: Flat Coated Retriever (n = 1), Boxer (n = 5), Standard Poodle (n = 2), German Shepherd cross (n = 6), German Shepherd/Golden Retriever cross (n = 12). One additional individual was excluded due to the complete absence of behavioural data. A population of 5575 dogs remained for further analysis.

In human studies, individuals with joint hypermobility are regarded as a specific clinical group associated with a range of psychological and psychiatric characteristics. As no such classification of joint hypermobility exists within the dog population, it was decided to use cluster analysis to identify suitable high and low joint hypermobility subpopulations using the PennHip Distraction Index (DI) as the measure of joint hypermobility. Two-step clustering was chosen, as it can automatically generate an optimal clustering solution. The analysis of the dataset of 5575 cases a model with four clusters. The silhouette measure was 0.6 (see Table 2 for a summary of the clusters, with mean DI scores).

Cases from the clusters with the highest (n = 409, 7.3% of total population) and lowest (n = 2153, 38.6% of the total population) mean DI were carried forward for further analysis. Cases with >10% missing data for the C-BARQ were excluded, leaving a total of 2381 cases (2002 high DI, 379 low DI) and any remaining missing C-BARQ data was replaced with the median. Scores for the sub-scales of the C-BARQ were calculated according to the authors directions.

Binary logistic regression (enter method. SPSS 22, IBM) was used to identify factors associated with membership of the high or low DI groups. To select the variables that would be included in the BLR model, sex, breed, and C-BARQ scores were compared between the high and low DI groups using appropriate Chi-square and parametric or non-parametric contrasts: Variables with p < 0.1 were included in the binary logistic regression analysis.

After variables that did not differ significantly between the two clusters at p < 0.1 were excluded, sex and C-BARQ sub-scale scores for “owner-directed aggression”, “familiar dog directed aggression”, “trainability”, “chasing” “non-social fear”, “touch sensitivity”, and “excitability” were included in the BLR model. The model passed omnibus tests of model coefficients. Nagelkerke R^2^ was 0.458 and overall classification accuracy was 89.2%. Table 2 summarises the findings of the binary logistic regression model, with odds ratios and confidence intervals for the included variables.

## Data Availability

Data will be made available upon request through the corresponding author.
